# Evaluation of Coated Urea Products as Supplemental Nitrogen Alternatives to Soybean Meal in Dairy Cow Diets

**DOI:** 10.3390/ani16213476

**Published:** 2026-11-03

**Authors:** Zhen Chen, Zhiqiang Cheng, Yuan Gao, Shiwei Yang, Xiaoqing Hong, Heng Wang, Xuan Hu, Kang Zhan, Yongjiu Huo, Miao Lin, Yimin Zhuang, Guoqi Zhao, Yihui Zhang

**Affiliations:** 1 College of Animal Science and Technology, Yangzhou University, Yangzhou 225009, China; cz18756682252@163.com (Z.C.); zqcheng1998@163.com (Z.C.);; 2 Shanghai Menon Animal Nutrition Technology Co., Ltd., Shanghai 201800, China; gaoyuannoone@163.com (Y.G.);; 3 Experimental Farm of Yangzhou University, Yangzhou University, Yangzhou 225009, China

**Keywords:** slow-release urea, in situ disappearance, rumen fermentation, supplemental nitrogen

## Abstract

Coated slow-release urea (SRU) may provide an alternative source of supplemental nitrogen in dairy cattle diets, but its ruminal release behavior can vary among formulations. This study compared three palm-oil-coated SRU products with different nominal release specifications using nonlactating, nonpregnant, rumen-cannulated Chinese Holstein cows. Three cows were used in an in situ experiment to characterize dry matter (DM) and urea disappearance from 0.5 to 12 h, and four cows were assigned to a 4 × 4 Latin square feeding trial. The control treatment consisted of a basal diet plus 500 g/d supplemental soybean meal (SBM), whereas the SRU treatments received the same basal diet plus 100 g/d SRU I, SRU II, or SRU III as approximately isonitrogenous supplemental nitrogen alternatives. SRU I consistently showed slower early DM and urea disappearance than SRU II and SRU III, whereas differences between SRU II and SRU III were less consistent across incubation times. Dietary treatment did not significantly affect DM, crude protein, or neutral detergent fiber intake, apparent total-tract digestibility, ruminal pH, total or individual volatile fatty acid concentrations, or hematological variables. Ruminal ammonia nitrogen differed among treatments (*p* = 0.04), with SRU III having a higher concentration than the control and SRU I and SRU II showing intermediate values. These results demonstrate formulation-dependent in situ disappearance patterns among coated SRU products and suggest that they can serve as alternative supplemental nitrogen sources under the conditions tested.

## Simple Summary

Urea provides non-protein nitrogen that ruminal microorganisms can use for microbial protein synthesis. Coating urea with palm oil is intended to slow its release, although release characteristics may differ among products. This study compared three coated slow-release urea products in non-lactating, non-pregnant dairy cows. SRU I showed slower early ruminal disappearance than SRU II and SRU III. In a short-term feeding trial, 100 g/day of coated urea was supplied as an approximately isonitrogenous supplemental nitrogen alternative to 500 g/day of supplemental soybean meal. Under the conditions tested, dietary treatment did not produce detectable differences in feed intake, apparent nutrient digestibility, most ruminal fermentation variables, or hematological measurements. SRU III resulted in a higher ruminal ammonia nitrogen concentration than the control. These findings show that coated urea can provide supplemental nitrogen in dairy cow diets, but they do not establish nutritional equivalence to soybean meal or demonstrate a practical advantage over conventional urea. Larger and longer-term studies are needed to evaluate its practical application.

## 1. Introduction

Efficient use of dietary nitrogen is a persistent challenge in dairy cattle nutrition because a substantial proportion of consumed nitrogen is not retained in body or milk protein and is ultimately excreted [1,2,3]. In the rumen, degradable nitrogen supports microbial protein synthesis when ammonia availability is synchronized with fermentable energy supply [1,4,5]. Conventional urea is rapidly hydrolyzed by microbial urease, and ammonia production can temporarily exceed microbial assimilation, increasing absorption of ammonia across the rumen wall and hepatic conversion to urea [6,7,8,9]. Strategies that moderate ruminal urea release may therefore improve control of nitrogen availability while reducing reliance on plant-derived protein ingredients.

Coated slow-release urea (SRU) products are designed to delay urea dissolution through physical or chemical coating systems [10,11,12,13]. However, published responses to SRU are not uniform. Effects on ruminal ammonia, nutrient digestibility, intake, and animal performance vary with product formulation, release characteristics, dietary composition, and inclusion level [10,12,13,14]. Recent work has also shown that SRU compounds can differ substantially in degradation behavior [12], indicating that results obtained with one formulation cannot necessarily be generalized to another. In addition, relatively few in vivo studies have directly compared multiple formulations produced on a common coating platform but designed for different release rates. Direct comparisons under the same dietary conditions, while linking formulation-specific ruminal release kinetics with animal-level fermentation and digestibility responses, are therefore useful for distinguishing coating-dependent effects from general effects of dietary urea supplementation.

Accordingly, the present study compared three coated SRU products with a soybean meal-based supplemental protein control. An in situ ruminal incubation experiment was used to characterize formulation-specific dry matter and urea disappearance kinetics, followed by a 4 × 4 Latin square feeding trial in nonlactating, nonpregnant Holstein cows to evaluate feed intake, apparent total-tract digestibility, ruminal fermentation, and hematological responses. Because conventional uncoated urea was not included, the study was designed to compare formulation-specific responses of the coated SRU products with an SBM-based control rather than to determine whether coating itself provides an advantage over conventional feed-grade urea. We hypothesized that the three coated SRU formulations would exhibit distinct ruminal release kinetics and that replacing supplemental soybean meal with coated SRU would maintain the measured digestive and physiological responses under approximately isonitrogenous conditions.

## 2. Materials and Methods

### 2.1. Experimental Materials

Three coated SRU formulations, designated SRU I, SRU II, and SRU III, were obtained from the R&D Center of Shanghai Menon Animal Nutrition Technology Co., Ltd. (Shanghai, China). All three formulations were manufactured using the same hot-melt fluidized-bed coating platform. Urea was used as the core material, and the nominal core-to-coating mass ratio was 88:12 (*w*/*w*). The coating material consisted mainly of palm oil and a proprietary hydrophilic release-modifying excipient. The release modifier accounted for 5%, 10%, and 15% (*w*/*w*) of the coating material in SRU I, SRU II, and SRU III, respectively. According to the manufacturer’s specifications, the nominal 1-h urea release rates of SRU I, SRU II, and SRU III were 5 ± 3%, 15 ± 3%, and 25 ± 3%, respectively, when tested in simulated rumen buffer (pH 6.8 ± 0.1, 39 °C). The finished products were free-flowing coated granules with a particle-size range of approximately 0.85–3.35 mm. The dry matter and urea contents of the three SRU products were determined using standard methods in the authors’ laboratory at Yangzhou University, independently of the manufacturer’s specifications. The dry matter contents of the experimental batches were 99.17%, 99.36%, and 99.15%, and the urea contents were 84.83%, 81.68%, and 81.70% for SRU I, SRU II, and SRU III, respectively. All basal-diet ingredients were purchased from Youxin Feed Co., Ltd. (Yancheng, China).

### 2.2. Animals, Housing, and Management

The experiment was conducted from July to October 2025 at the Dairy Cattle Experimental Farm of the Animal Nutrition and Feed Engineering Research Center, Yangzhou University (Gaoyou, Yangzhou, Jiangsu, China). Four clinically healthy, nonlactating, nonpregnant, multiparous Chinese Holstein cows (approximately 5 years of age) fitted with permanent ruminal cannulas were used. The mean body weight at the beginning of the study was 647.3 ± 22.1 kg. The cows were not bred during the experiment, and no calving occurred. Nonlactating, nonpregnant cows were selected because this physiological state minimizes confounding from lactation and pregnancy, which can affect nitrogen metabolism, feed intake, and rumen function, and because the number of available rumen-cannulated animals was limited. Three of the four cows used in Experiment 2 had also been used in Experiment 1 to reduce the number of animals required and to maintain consistency of ruminal conditions. Cows were housed individually in tie stalls with free access to water. All procedures were approved by the Institutional Animal Care and Use Committee of Yangzhou University (Approval No. IACUC-Su-2026-0029; approved on 20 May 2025).

### 2.3. Experimental Design

#### 2.3.1. Experiment 1: In Situ Ruminal Disappearance

Three rumen-cannulated cows were used to evaluate the ruminal release kinetics of the three SRU formulations using the in situ nylon-bag technique. Before incubation, cows were adapted to the basal diet for 7 day and were offered approximately 15 kg DM/cow per day in three meals at 08:00, 14:00, and 22:00 h. In situ incubations were then conducted over 5 day. Nylon bags (8 × 12 cm; 50 μm pore size) were prewashed, dried at 65 °C for 24 h to constant weight, and weighed. Approximately 5 g of each SRU formulation was placed into each bag. All three formulations were incubated in each cow. For each cow, formulation, and incubation time, three bags were incubated as technical replicates, yielding nine bags per formulation per time point across the three cows. Bags were placed in the rumen immediately before the morning feeding and recovered after 0.5, 1, 2, 4, 8, and 12 h of incubation. After removal, bags were placed loosely in a 20-L plastic bucket containing approximately 15 L of tap water and gently agitated by hand for 1 min. This washing procedure was repeated three times with fresh water. Bags were then dried at 65 °C for 48 h to constant weight and weighed. Urea contents of the original SRU samples and ground residues were determined using the diacetyl monoxime colorimetric method [15].

Dry matter (*DM*) *disappearance* was calculated as:

(1) *DM disappearance* (%) = 100 × (*initial DM* − *residual DM*)/*initial DM*

*Urea disappearance* was calculated as:

(2) *Urea disappearance* (%) = 100 × (*initial urea* − *residual urea*)/*initial urea*

Because urea is highly water soluble, disappearance from the nylon bags was interpreted as in situ release and solubilization rather than as direct microbial degradation. No zero-time washing-loss measurement was performed; therefore, the present experiment focused on the observed disappearance profiles from 0.5 to 12 h without fitting conventional degradation-fraction parameters or calculating effective degradability.

#### 2.3.2. Experiment 2: 4 × 4 Latin Square Feeding Trial

Experiment 2 used a 4 × 4 Latin square design in which four rumen-cannulated cows were assigned to four treatment sequences across four 10-d periods. Each period comprised 7 d of dietary adaptation followed by 3 d of sample collection. All cows received the same basal diet. The control treatment consisted of the basal diet plus 500 g/cow per day of soybean meal. The three SRU treatments consisted of the basal diet plus 100 g/cow per day of SRU I, SRU II, or SRU III, respectively, replacing the supplemental soybean meal on an approximately isonitrogenous basis according to the urea content of each product. Diets were prepared fresh daily and offered ad libitum in three meals at 08:00, 14:00, and 22:00 h, with feed allowance adjusted to maintain approximately 5–10% orts. Water was available ad libitum. The total Latin square lasted 40 d, and the cow was the experimental unit.

### 2.4. Sample Collection and Measurements

#### 2.4.1. Feed Intake and Feed Sampling

Daily feed intake was calculated as the difference between feed offered and orts after 24 h. Samples of corn silage, oat hay, and total mixed ration (TMR) were collected weekly, pooled within each period, and stored at −20 °C until analysis. Concentrate samples were collected at preparation and stored at −20 °C until analysis. The nutrient composition presented in Table 1 represents the calculated composition of the formulated basal diet.In contrast, the nutrient intakes for Experiment 2 were derived from the actual feed intake during each experimental period together with the laboratory-determined nutrient concentrations of the corresponding feed samples. 

#### 2.4.2. Total Fecal Collection and Apparent Nutrient Digestibility

A 48-h total fecal collection was conducted from 10:00 h on day 8 to 10:00 h on day 10 of each period to estimate apparent total-tract nutrient digestibility. The 48-h collection period was based on a previously described procedure for tie-stall dairy cows, in which a comparable 48-h total fecal collection was used for repeated digestibility and nitrogen-balance measurements [16]. All feces excreted by each cow during the collection interval were manually and quantitatively collected, weighed, and thoroughly mixed. Representative subsamples (approximately 10% of fresh weight) were obtained proportionally from the homogenized fecal material and stored at −20 °C until analysis. Apparent nutrient digestibility was calculated from nutrient intake and fecal nutrient output.

#### 2.4.3. Blood and Rumen Fluid Sampling

On day 10 of each period, blood samples were collected from the coccygeal vein 2 h after the morning feeding. Blood for hematological analysis was collected into EDTA tubes. Rumen fluid was collected 2 h after the morning feeding because this time point was selected as a standardized early postprandial sampling point intended to characterize early changes in ruminal nitrogen availability following feed consumption. This sampling scheme was designed for standardized treatment comparison rather than characterization of the complete diurnal fermentation profile. Approximately 100 mL of rumen fluid was collected and filtered through four layers of cheesecloth. For volatile fatty acid (VFA) and ammonia nitrogen (NH_3_-N) analysis, 1 mL of rumen fluid was mixed with 0.2 mL of 20% metaphosphoric acid. Rumen pH was measured immediately using a portable pH meter (Sartorius, Göttingen, Germany), and the remaining samples were stored at −20 °C until analysis.

### 2.5. Laboratory Analyses

Dry matter (DM), crude protein (CP), ether extract (EE), and ash were determined according to AOAC International methods [17]. Neutral detergent fiber (NDF) and acid detergent fiber (ADF) were determined using the detergent procedures of Van Soest et al. [18]. Rumen fluid samples were centrifuged at 12,000× *g* for 10 min at 4 °C and filtered through a 0.22-μm membrane. The VFAs, including acetate, propionate, isobutyrate, butyrate, isovalerate, valerate, and caproate, were quantified by gas chromatography (GC-3800, Shanghai Kechuang Chromatography Instruments Co., Ltd., Shanghai, China) using a DB-FFAP capillary column (30 m × 0.320 mm internal diameter × 0.25 μm film thickness; part no. 123-3232, Agilent Technologies, Inc., Santa Clara, CA, USA) with crotonic acid as the internal standard. The injector, column, and detector temperatures were 200 °C, 110 °C, and 200 °C, respectively. Nitrogen was used as the carrier gas at 50 mL/min, and the injection volume was 1 μL. Ruminal NH_3_-N concentration was determined using the phenol-hypochlorite colorimetric method described for rumen fluid by Broderick and Kang [19]. Hematological parameters were measured using an automated veterinary hematology analyzer (VH50, Shenzhen Mindray Bio-Medical Electronics Co., Ltd., Shenzhen, China).

### 2.6. Statistical Analysis

For Experiment 1, the three parallel bags within each cow, formulation, and incubation time were treated as technical replicates and averaged before statistical analysis; therefore, cow was considered the experimental unit. Disappearance data were analyzed using a repeated-measures model with SRU formulation, incubation time, and their interaction as fixed effects and cow as the blocking factor. Because significant formulation × incubation time interactions were detected, formulation effects were subsequently evaluated within each incubation time using a randomized complete block model with cow as the block. Pairwise comparisons among formulations were adjusted using Tukey’s method. Model-derived SEM values corresponding to these comparisons are reported. Statistical significance was declared at *p* ≤ 0.05.

For Experiment 2, period-level data were analyzed using the MIXED procedure of SAS 9.2. The model included treatment and period as fixed effects and cow as a random effect:

(3) *Yijk = μ + Ti + Pj + Ck + εijk*

where *Yijk* is the dependent variable, *μ* is the overall mean, Ti is the fixed effect of treatment (*i* = 1–4), *Pj* is the fixed effect of period (*j* = 1–4), *Ck* is the random effect of cow (*k* = 1–4), and *εijk* is the residual error. Model assumptions were assessed using residual plots, the Shapiro–Wilk test for residual normality, and Levene’s test for homogeneity of variance. When the overall treatment effect was significant, pairwise comparisons were adjusted using Tukey’s method. Statistical significance was declared at *p* ≤ 0.05. Given the small number of experimental units, an approximate sensitivity analysis indicated minimum detectable between-treatment differences of 1.58 kg/d for DM intake, 3.49 percentage points for DM digestibility, 17.22 mM for TVFA, and 9.32 mg/dL for ruminal NH_3_-N at 80% power and a two-sided α of 0.05.

## 3. Results

### 3.1. In Situ Dry Matter Disappearance

Ruminal DM disappearance increased with incubation time in all three SRU formulations, and the magnitude of disappearance differed among formulations across the incubation period (Table 2). At 0.5 h, DM disappearance was lowest for SRU I (33.48%), intermediate for SRU II (65.03%), and highest for SRU III (78.85%). SRU I remained lower than SRU II and SRU III throughout the 12-h incubation. Differences between SRU II and SRU III were most evident during the early incubation period and diminished at later time points. At 12 h, DM disappearance reached 82.82%, 88.37%, and 90.33% for SRU I, SRU II, and SRU III, respectively.

### 3.2. In Situ Urea Disappearance

Urea disappearance increased rapidly during the initial incubation period and differed among formulations over time (Table 3). At 0.5 h, disappearance was 34.76%, 75.43%, and 91.01% for SRU I, SRU II, and SRU III, respectively. SRU I consistently showed lower urea disappearance than the other formulations. During the early incubation period (0.5–2 h), SRU III also showed significantly greater urea disappearance than SRU II, whereas SRU II and SRU III did not differ significantly at 4, 8, or 12 h. By 12 h, urea disappearance reached 93.77%, 99.63%, and 100.00% for SRU I, SRU II, and SRU III, respectively.

### 3.3. Feed Intake and Apparent Total-Tract Digestibility

Treatment did not affect DM, CP, or NDF intake (Table 4). Dry matter intake ranged from 14.16 to 14.71 kg/d. Apparent total-tract digestibility of DM, CP, and NDF also did not differ among treatments.

**Table 4:** Effects of coated urea products on feed intake and apparent total-tract digestibility in dairy cows.

Item ^1^	Control	SRU I	SRU II	SRU III	SEM	*p*-Value
DM intake (kg/d)	14.45	14.23	14.16	14.71	0.40	0.521
CP intake (kg/d)	1.68	1.68	1.70	1.72	0.11	0.557
NDF intake (kg/d)	5.97	5.96	5.96	6.17	0.19	0.593
DM digestibility (%)	65.1	64.7	65.3	65.5	0.88	0.759
CP digestibility (%)	72.7	73.4	72.9	72.1	1.17	0.325
NDF digestibility (%)	56.4	56.6	55.8	54.2	1.55	0.402

^1^ DM = dry matter; CP = crude protein; NDF = neutral detergent fiber; SRU = slow-release urea; SEM = standard error of the mean. Cow was the experimental unit (*n* = 4). The analyzed CP concentrations of the treatment TMRs were 11.59%, 11.83%, 11.97%, and 11.70% of DM for the Control, SRU I, SRU II, and SRU III treatments, respectively; the corresponding analyzed NDF concentrations were 41.29%, 41.85%, 42.10%, and 41.92% of DM, respectively. CP and NDF intakes were calculated using the actual DM intake and the corresponding analyzed nutrient concentrations.

### 3.4. Ruminal Fermentation Characteristics

Ruminal pH ranged from 6.00 to 6.07 and did not differ among treatments (Table 5). Total volatile fatty acid (TVFA) and individual VFA concentrations were also not affected by treatment. Ruminal NH_3_-N concentration differed among treatments (*p* = 0.04); SRU III had a greater concentration than the control, whereas SRU I and SRU II were intermediate and did not differ from either the control or SRU III.

### 3.5. Hematological Responses

No significant differences were observed among treatments in leukocyte, erythrocyte, or platelet profiles (Table 6). Leukocyte-related parameters, including white blood cell count, neutrophil and lymphocyte percentages, remained within similar ranges across all groups. Erythrocyte indices, including red blood cell count, hemoglobin concentration, and hematocrit, were not affected by dietary treatment. Similarly, platelet-related parameters, including platelet count, mean platelet volume, and plateletcrit, showed no significant differences among treatments.

## 4. Discussion

The principal finding of the present study was that coated SRU formulations produced using the same coating platform nevertheless exhibited formulation-dependent differences in ruminal disappearance, whereas replacing supplemental SBM with these products produced relatively limited changes in the measured digestive, fermentative, and hematological responses. The differences among SRU formulations were most pronounced during the early phase of ruminal incubation. SRU I consistently showed slower DM and urea disappearance, whereas SRU II and SRU III exhibited substantially greater early disappearance. However, the separation between SRU II and SRU III was not maintained at all incubation times, particularly for urea disappearance. Thus, the present results support formulation-dependent release patterns rather than three completely distinct kinetic phenotypes. These findings further indicate that slow-release urea should not be considered a uniform category of feed ingredient, because its ruminal release behavior may depend strongly on the physicochemical characteristics of the individual formulation [11,12].

The observed differences can reasonably be related to variation in coating design. All three products were manufactured using the same palm-oil-based hot-melt fluidized-bed coating process, but the proportion of the proprietary hydrophilic release-modifying excipient differed among formulations. Such differences would be expected to alter water penetration into the coating matrix and the subsequent dissolution and release of the highly water-soluble urea core [12,20]. The relative ranking observed during in situ incubation was generally consistent with the intended release classes, with SRU I showing the slowest early disappearance and SRU III the most rapid. Nevertheless, the nominal release specifications were obtained in simulated rumen buffer, whereas the present measurements were generated using an in situ nylon-bag procedure; therefore, the absolute values obtained with the two methods should not be directly compared. In particular, disappearance from the nylon bag reflects coating disruption, solubilization, and physical loss from the bag rather than direct microbial degradation of urea. Moreover, because no zero-time washing-loss control was performed, very early loss attributable solely to handling and washing could not be distinguished from rapid release during the initial incubation period. Accordingly, the present in situ data should be interpreted as comparative disappearance profiles under the specified incubation and washing conditions rather than as estimates of conventional feed degradation fractions [21].

The ruminal NH_3_-N response provides additional evidence that these differences in release kinetics affected the temporal availability of N in the rumen. Urea entering the rumen is rapidly hydrolyzed by microbial ureases to ammonia, which can subsequently be incorporated into microbial protein when sufficient fermentable energy and other nutrients are available [20,22]. A rapid supply of urea-N can therefore transiently increase ruminal NH_3_-N when ammonia production exceeds microbial capture or removal from the rumen [6]. In the present study, SRU III, which exhibited the fastest early urea disappearance, also produced the highest NH_3_-N concentration at 2 h after feeding, whereas SRU I and SRU II produced intermediate concentrations. This correspondence between the in situ release phenotype and the in vivo NH_3_-N response strengthens the interpretation that formulation-dependent release rate influenced early postprandial N availability. However, ruminal NH_3_-N concentration alone is not a measure of microbial N capture or whole-animal N efficiency. Because microbial protein synthesis, ammonia absorption, urea recycling, urinary N excretion, and total N balance were not quantified, the present results should not be interpreted as demonstrating improved N synchronization or N-use efficiency for any individual SRU formulation [3,23].

In contrast to NH_3_-N, ruminal pH and total and individual VFA concentrations did not differ detectably among treatments at 2 h after feeding, and feed intake and apparent total-tract digestibility were also similar. This pattern is broadly consistent with previous evidence showing that moderate replacement of true-protein supplements with SRU often has limited effects on DMI and nutrient digestibility, although ruminal ammonia is more responsive to the form and supply of dietary N [10,14,24]. A network meta-analysis of studies in lactating dairy cattle similarly found generally small effects of feed-grade or slow-release urea on intake and digestibility relative to true-protein controls, while ruminal NH_3_-N increased with urea supplementation [25]. The relatively stable VFA profile observed here may reflect the fact that the treatments primarily altered the source and release pattern of supplemental N while maintaining a common basal diet and broadly similar fermentable substrate supply. Nevertheless, because ruminal fermentation was assessed at only one post-feeding time, these data cannot exclude transient treatment differences earlier or later in the feeding cycle. Likewise, the relatively short experimental periods and 48-h fecal collection warrant interpreting the digestibility results as an absence of detected treatment effects under the present conditions rather than evidence of nutritional equivalence across longer production periods [13].

From a feeding perspective, the maintenance of intake, apparent digestibility, and the measured fermentation characteristics suggests that coated urea can provide part of the supplemental N otherwise supplied by SBM without readily detectable disruption of ruminal function under the conditions tested. This observation agrees with previous dairy-cow studies in which partial substitution of SBM with SRU maintained most production or fermentation responses, although the effects on N metabolism have not been uniformly beneficial and may depend on diet composition and the release characteristics of the SRU source. This distinction is important when considering the practical value of SRU. Replacing a portion of true-protein supplements with non-protein N could reduce reliance on conventional protein ingredients and potentially increase flexibility in ration formulation. Such substitution may also have implications for the use of human-edible protein resources and the environmental N burden of dairy production [26,27]. However, these potential sustainability benefits cannot be demonstrated from the present experiment because feed-source life-cycle impacts, N excretion, N-use efficiency, milk production, and economic responses were not measured. Consequently, the present findings support the technical feasibility of partial replacement of supplemental SBM rather than demonstrating an environmental or economic advantage [28].

Hematological variables did not differ significantly among dietary treatments, indicating that no treatment-associated changes were detected in the measured blood-cell indices during the experimental periods. Published reference intervals for bovine hematological variables vary considerably according to animal population, physiological status, analytical platform, and laboratory-specific methodology. For example, previously reported reference intervals for adult cattle span approximately 4.9–10.0 × 10^12^/L for erythrocytes, 21–38% for hematocrit, 84–140 g/L for hemoglobin, 4.9–13.3 × 10^9^/L for leukocytes, and 160–800 × 10^9^/L for platelets, depending on the reference population and analytical system [29,30,31]. In the present study, several erythrocyte-related treatment means were close to or slightly outside some published reference intervals, whereas other hematological variables broadly overlapped reported bovine ranges. These comparisons should be interpreted cautiously because the published intervals were generated from different cattle populations and hematology analyzers. Accordingly, our findings are interpreted primarily as evidence of an absence of detectable treatment-related hematological changes rather than as proof that every measured value was within a universal physiological reference range or that the treatments were comprehensively metabolically safe [13].

Several features of the experimental design define the scope of these conclusions. Although the 4 × 4 Latin square design involving four nonlactating dairy cows allowed within-animal comparisons among the four dietary treatments, the small sample size limited the statistical power to detect subtle treatment effects. Furthermore, nonlactating and lactating dairy cows differ substantially in nitrogen metabolism, feed intake regulation, and rumen function. Therefore, these findings should not be extrapolated to lactating dairy cows without further validation. In addition, the 10-d experimental periods, 48-h fecal collection, and single post-feeding rumen and blood sampling provide limited information on longer-term adaptation and diurnal N dynamics; future studies should therefore collect rumen fluid at multiple post-feeding time points to better characterize diurnal fermentation patterns. The absence of direct measurements of microbial protein synthesis, N balance, urea recycling, and N excretion further prevents mechanistic conclusions regarding N-use efficiency. Finally, only one inclusion level was evaluated and conventional feed-grade urea was not included; thus, the present study can distinguish release behavior among the tested coated formulations and compare their responses with supplemental SBM, but cannot establish whether coating itself conferred an advantage over uncoated urea. Future studies should therefore evaluate these formulation-specific release profiles in lactating cows over longer experimental periods, with repeated postprandial sampling and direct measurements of microbial N synthesis, blood urea, urinary and fecal N excretion, milk N output, and whole-animal N balance. Such studies would determine whether the formulation-dependent release patterns observed here translate into meaningful improvements in N utilization, productive performance, or environmental outcomes.

## 5. Conclusions

The tested coated SRU products showed formulation-dependent in situ disappearance patterns, with SRU I exhibiting slower early DM and urea disappearance than SRU II and SRU III. In the 4 × 4 Latin square trial, supplying 100 g/d SRU as an approximately isonitrogenous supplemental nitrogen alternative to 500 g/d supplemental soybean meal did not significantly affect feed intake, apparent total-tract digestibility, ruminal pH, VFA concentrations, or hematological variables, whereas SRU III resulted in the highest ruminal NH_3_-N concentration. These findings support formulation-specific evaluation of coated urea as an alternative supplemental nitrogen source, but do not establish nutritional equivalence to soybean meal or an advantage over uncoated urea.

## Figures and Tables

**Table 1:** Ingredient composition and calculated nutrient composition of the basal diet (%, DM basis).

Ingredients	Inclusion (%)
Corn	19.20
Barley	5.20
Cottonseed meal	3.00
Distillers dried grains	4.50
Soybean meal ^1^	3.50
Corn gluten meal	1.80
Limestone	0.32
Premix ^2^	1.20
Sodium chloride	0.30
Dicalcium phosphate	0.55
Sodium bicarbonate	0.35
Oat hay	8.50
Corn silage	51.58
Nutrient composition ^3^	
CP	13.20
EE	3.64
NDF	39.86
ADF	21.25
Ash	7.28
Calcium	0.78
Phosphorus	0.40
NE_L_(MJ/kg) ^4^	5.95

^1^ The soybean meal listed here (3.50% of DM) was included in the common basal diet and was fed to all treatments. It was separate from the 500 g/d supplemental soybean meal provided only in the control treatment; the SRU treatments instead received 100 g/d of the respective coated urea product as an approximately isonitrogenous supplemental nitrogen source. ^2^ Each kilogram of premix contained: vitamin A, 300,000 IU; vitamin D, 385,000 IU; vitamin E, 1455 IU; niacin, 550 mg; Cu, 770 mg; Mn, 930 mg; Fe, 1200 mg; Zn, 3600 mg; Se, 21 mg; I, 50 mg; and Co, 12 mg. ^3^ The nutrient composition presented in Table 1 represents calculated values based on the formulated basal diet and was used to describe the intended dietary formulation. ^4^ Estimated based on the NASEM (2021).

**Table 2:** In situ dry matter disappearance (%) of coated urea products.

Incubation Time (h)	SRU I	SRU II	SRU III	SEM ^1^	*p*-Value
0.5	33.48 ^c^	65.03 ^b^	78.85 ^a^	1.6	<0.001
1	40.16 ^c^	74.19 ^b^	81.84 ^a^	0.93	<0.001
2	52.28 ^c^	79.39 ^b^	86.82 ^a^	0.99	<0.001
4	74.61 ^b^	85.25 ^a^	89.24 ^a^	1.17	0.002
8	81.25 ^b^	87.19 ^a^	89.52 ^a^	0.57	0.001
12	82.82 ^b^	88.37 ^a^	90.33 ^a^	0.93	0.01

^1^ SEM = standard error of the mean derived from the cow-blocked model. Cow was the experimental unit (*n* = 3); triplicate bags within cow were averaged as technical replicates. ^a–c^ Means within a row with different superscript letters differ significantly according to Tukey-adjusted comparisons (*p* < 0.05).

**Table 3:** In situ urea disappearance (%) of coated urea products.

Incubation Time (h)	SRU I	SRU II	SRU III	SEM ^1^	*p*-Value
0.5	34.76 ^c^	75.43 ^b^	91.01 ^a^	1.34	<0.001
1	43.64 ^c^	83.74 ^b^	93.05 ^a^	1.43	<0.001
2	57.88 ^c^	90.05 ^b^	97.63 ^a^	1.28	<0.001
4	83.84 ^b^	95.72 ^a^	98.75 ^a^	1.36	0.003
8	92.06 ^b^	97.62 ^a^	99.93 ^a^	0.77	0.005
12	93.77 ^b^	99.63 ^a^	100.00 ^a^	0.93	0.015

^1^ SEM = standard error of the mean derived from the cow-blocked model. Cow was the experimental unit (*n* = 3); triplicate bags within cow were averaged as technical replicates. ^a–c^ Means within a row with different superscript letters differ significantly according to Tukey-adjusted comparisons (*p* < 0.05).

**Table 5:** Effects of coated urea products on ruminal fermentation characteristics in dairy cows.

Item	Control	SRU I	SRU II	SRU III	SEM	Treatment *P*	Period *P*
pH	6.05	6.04	6.07	6.00	0.101	0.966	0.678
NH_3_-N ^1^, mg/dL	18.85 ^b^	23.11 ^ab^	25.91 ^ab^	31.76 ^a^	2.352	0.04	0.337
Acetate, mM	81.33	81.21	84.17	75.06	4.197	0.521	0.399
Propionate, mM	27.43	25.46	24.11	24.05	1.004	0.157	0.549
Isobutyrate, mM	0.727	0.754	0.720	0.667	0.039	0.496	0.485
Butyrate, mM	12.93	13.73	12.51	11.21	0.513	0.064	0.222
Isovalerate, mM	1.76	1.89	1.629	1.582	0.101	0.235	0.599
Valerate, mM	1.503	1.468	1.348	1.425	0.098	0.718	0.97
Caproate, mM	0.608	0.571	0.533	0.497	0.083	0.803	0.571
TVFA, mM	126.30	125.09	125.01	114.49	4.347	0.283	0.294

^1^ NH_3_-N = ammonia nitrogen; TVFA = total volatile fatty acids; SRU = slow-release urea; SEM = standard error of the mean. Cow was the experimental unit (*n* = 4). Within the same row, means with different superscript letters (^a^, ^b^) differ significantly (*p* < 0.05), whereas means sharing at least one superscript letter do not differ significantly, according to Tukey’s test.

**Table 6:** Effects of coated urea products on hematological parameters in dairy cows.

Item ^1^	Control	SRU I	SRU II	SRU III	SEM	Treatment *p*	Period *p*
Leukocyte profile							
WBC (10^9^/L)	5.15	5.54	6.9	7.18	0.557	0.105	0.537
Neutrophils (%)	45.53	47.1	41.58	45.55	6.219	0.93	0.909
Lymphocytes (%)	39.33	39.75	43.9	39.03	6.239	0.936	0.927
Erythrocyte profile							
RBC (10^12^/L)	4.38	4.47	4.37	5.07	0.342	0.471	0.249
HGB (g/L)	82.25	87.75	82.75	95.5	7.324	0.583	0.373
HCT (%)	23.03	23.78	22.6	26.7	2.084	0.544	0.341
Platelet profile							
PLT (10^9^/L)	241.5	194.25	298.5	289.25	50.421	0.49	0.383
MPV (fL)	8.18	8.18	7.83	7.9	0.341	0.832	0.434
PCT (%)	0.193	0.156	0.236	0.226	0.035	0.433	0.405

^1^ WBC = white blood cell count; RBC = red blood cell count; HGB = hemoglobin; HCT = hematocrit; PLT = platelet count; MPV = mean platelet volume; PCT = plateletcrit; SRU = slow-release urea; SEM = standard error of the mean. Cow was the experimental unit (*n* = 4).

## Data Availability

Upon reasonable request, the data presented in this study are available from the corresponding authors.
